# Intention to use long-acting and permanent contraceptive methods and associated factors in health institutions of Aksum Town, North Ethiopia

**DOI:** 10.1186/s13104-019-4769-z

**Published:** 2019-11-09

**Authors:** Hailay Syum, Gizienesh Kahsay, Teklehaymanot Huluf, Berhe Beyene, Hadgu Gerensea, Gebreamlak Gidey, Haftom Desta, Mebrahtu Abay, Haben Nuguse, Kebede Haile

**Affiliations:** 1 School of Public Health, College of Health Science and Comprehensive Specialized Hospital, Aksum University, Aksum, Ethiopia; 2 School of Nursing, College of Health Science and Comprehensive Specialized Hospital, Aksum University, Aksum, Ethiopia; 3 Department of Midwifery, College of Health Science and Comprehensive Specialized Hospital, Aksum University, Aksum, Ethiopia; 4 Department of Psychiatry Nursing, College of Health Science and Comprehensive Specialized Hospital, Aksum University, Aksum, Ethiopia; 5 School of Medicine, College of Health Science and Comprehensive Specialized Hospital, Aksum University, Aksum, Ethiopia

**Keywords:** LAPMs, Intention to use, Factors, Axum, Ethiopia

## Abstract

**Objective:**

In Ethiopia, the majority of married women practice predominantly short-acting contraceptive methods. Therefore this study aims to assess intention to use LAPMs and its determinants among short-acting users in Health Institutions of Aksum Town, North Ethiopia.

**Results:**

Prevalence of intention to use LAPMs was 52.1% (95% CI 47.4–57.0). Good knowledge on LAPMs [AOR = 2.15; 95% CI (1.29, 3.56)], positive attitude towards LAPMs [AOR = 3.41; 95% CI (1.99, 5.85)], 18–24 years of age [AOR = 3.18; 95% CI (1.30, 7.79)], being primary school in educational level [AOR = 0.34; 95% CI (0.14, 0.78)], decision on the number of children jointly with partner [AOR = 2.05; 95% CI (1.01, 4.18)], having more than two children [AOR = 10.67; 95% CI (1.29, 88.31)], and no [AOR = 10.21; 95% CI (3.10, 33.58)] and one [AOR = 4.70; 95% CI (1.68, 13.13)] extra number of children desired were factors significantly associated with having intention to use LAPMs compared to their counterparts. The intention to use LAPMs was low. Therefore, appropriate information, education and communication strategies must be designed to raise awareness and change the negative attitude of the community on LAPMs.

## Introduction

Family planning (FP) enables couples to determine whether, when, and how often to have children. Thereby it benefits everyone; women are healthier, more prosperous and have greater opportunities to pursue education and careers. Their children are stronger, better nourished and more successful in school. Families and communities can invest more in education and health care. Poverty is reduced; lives are saved [1, 2].

To envision the health benefits, evidence suggests that meeting the need for modern contraception among women in the developing region could prevent 1.6 million newborn deaths and 170,000 maternal deaths each year [3]. Therefore, it is very important to meet the FP need of the population and provide universal access to a full range of safe and reliable FP methods [1–3].

Notwithstanding the strikingly well-known and fascinating benefits, more than 225 million (26%) women who want to avoid pregnancy in a developing world still lack access to effective methods of contraception [3]. The FP need for 53% of the married women in Africa is unmet, which is two times higher than the average unmet need in the developed world [4, 5]. Even among the vast majority of the FP users in the developing world, the method is short-acting, which is less effective [1, 6, 7]. A total of 289,000 women per 100,000 live births per year and more than 7000 newborns per day die globally from very preventable problems that could have been deterred markedly through FP. Developing countries, mainly sub-Saharan Africa and South Asia, accounted for 80% of these deaths [3–5, 8, 9].

In Ethiopia, the unmet need for FP is 25%. Furthermore, the majority of the FP users in Ethiopia use short-acting methods of contraception. The long-acting and permanent methods of contraception, the most effective in preventing pregnancy, remained least used [10].

Intentions to perform a behavior translate into actual behavior when environmental obstacles can be overcome and individuals have skills to perform the behavior [11]. Hence, the intention to use LAPMs is the most important determinant of using the method [12, 13]. However, different individual and environmental factors may affect intention to use LAPMs. As indicated by some studies conducted elsewhere; socio-demographic characteristics, reproductive health characteristics, knowledge, attitude, myths, and misconceptions may affect it [14–19].

Given the prevailing low utilization of LAPMs in Ethiopia, an analysis of use intention and a critical assessment of the underlying factors are important. Because the generated inputs would have an imperative role in designing effective programs to solve the prevailing low level of LAPMs use. However, little has been documented so far in the aforementioned issue in Ethiopia. Thus, this study was conducted to assess the intention to use LAPMs and its determinants among married women of reproductive age in Axum Town, Northern Ethiopia.

## Main text

### Methods

#### Study design, period and setting

The study area was Aksum Town. Aksum is found in the Central zone of Tigray regional state located 1025 km north of Addis Ababa. Aksum has a total population of 60,706. The number of the female population in Aksum is 30,657 of these 12,214 are women in the reproductive age group. Aksum has one referral hospital, two governmental health centers, and one local NGO clinic; all of them provide maternal health services. An institution-based cross-sectional study was conducted. The study was conducted from February to March 2016.

#### Source population

The source population was all women who are short-acting contraceptive users of health institutions of Aksum Town.

#### Study population

The study population was all women who are short-acting contraceptive users that visited health institutions of Aksum Town during the study period.

#### Inclusion and exclusion criteria

##### Inclusion criteria

All women who are on short-acting contraceptive methods in health institutions of Aksum Town.

##### Exclusion criteria

No exclusion criteria.

#### Sample size determination

The sample size was calculated using a single population proportion formula with the assumption margin of error is 5%, and the confidence level is 95%. The prevalence of intention to use LAPMs was used 48.4% [15]. The calculated sample size was 384. Adding none response rate 5% the final sample size used was 403.

#### Sampling procedure

The total sample size was divided proportionally to the four health institutions based on their client flow. The proportionally allocated sample size was Aksum St. Merry Hospital = 216, Aksum Health Center = 105, Millennium Health Center = 49, and Family Guidance Association of Ethiopia (FGAE) clinic = 33. The study participants were taken by systematic random sampling, every 2nd of the clients (k = 2.1) and the 1st client was selected by simple random sampling from 1 and 2.

#### Operational definition

##### Intention to use LAPMs

Desire to use long-acting and permanent contraception methods as reported by the study participant.

##### Knowledge about LAPMs

Ten awareness questions (with ‘Yes/No’ answers) were included [19, 20]. The score is calculated by adding values given to each of the ten questions. Then, it was categorized as low knowledge and good knowledge based on the mean score.

##### Attitude about LAPMs

Ten questions (with ‘Yes/No’ answers) were included. An attitude score was computed using the same procedure that was used to compute the knowledge score. Then, it was categorized as a negative attitude and Positive attitude based on the mean score.

#### Data collection procedure

A structured pre-tested questionnaire, prepared via reviewing of literature, was used. The questionnaire was initially prepared in English and translated into Tigrigna (local language) and again retranslated back to English to check for any inconsistencies or distortions in the meaning of words and concepts. Face to face interview was the technique of data collection (Additional file 1). For administering the interview four diploma nurses, data collectors, were recruited.

#### Data quality control

To assure data quality the data collection tool was translated into the local language. The recruited data collectors were trained for 2 days on the tool, objectives, confidentiality of the study. The structured questionnaire was pre-tested on 5% of the sample size outside of the study area, in Laelay Maychew district. Close supervision was undertaken during data collection.

#### Data processing and analysis

All collected data were entered into EPI-info version 7 and transferred to SPSS version 20 statistical software for its analysis. Data were cleaned and coded for completeness and consistency. A binary logistic regression model was used for analysis. Factors associated with intention to use LAPMs at bivariable analysis were identified and the variables with a P-value of 0.20 and less were fit to logistic model for multivariable analysis to determine relative prediction level of independent variables to the outcome variable. A P-value less than 0.05 at 95% confidence interval was considered as statistically significant. Model goodness-of-fit was checked by the Hosmer–Lemeshow test.

### Results

#### Socio-demographic characteristics

A total of 384 women participated in the study with a response rate of 95.3%. The ages of respondents were ranged from 18 to 45 years with the mean age of 30.3 (SD ± 6.8) years, and about one-third of the women 121 (31.5%) were in the age group ≥ 35 years. Almost all of the respondents 381 (99.2%) were Tigray in Ethnicity and 370 (96.4%) were Orthodox Christians in religion (Table 1).

**Table 1:** Socio-demographic characteristics of women utilizing short acting family planning service in health institutions, Axum town, northern Ethiopia, March, 2016 (n = 384)

Variables	Frequency	Percent
Age in years		
18–24	87	22.7
25–34	176	45.8
≥ 35	121	31.5
Religion		
Orthodox	370	96.4
Muslim	14	3.6
Household size		
1–4	259	67.4
5–10	125	32.6
Marital status		
Married	358	93.2
Single	26	6.8
Mothers’ education		
No formal education	65	16.9
Primary school	81	21.1
Secondary school	141	36.7
Post secondary	97	25.3
Mothers’ occupation		
House wife	190	49.5
Private business	56	14.6
Employed	93	24.2
Unemployed	45	11.7

#### Reproductive characteristics

The mean ideal desired number of children of the participant was 2 (± 1.2 SD) and the decision on the number of children to have was decided jointly by the husband and wife in 317 (82.6%) of the participants (Table 2).

**Table 2:** Reproductive characteristic of women utilizing short acting family planning service in health institutions, Axum town, northern Ethiopia, March, 2016 (n = 384)

Variables	Frequency	Percent
History of abortion		
Yes	51	13.3
No	333	86.7
Number of abortions		
0	333	86.7
1–2	51	13.3
Number of children alive		
< 2	260	67.7
> 2	124	32.3
Desire for more child		
Yes	328	85.4
No	56	14.6
Number of desired children		
0	56	14.6
1	77	20.1
2	122	31.8
3	80	20.8
4–6	49	12.8

#### The magnitude of intention to use LAPMs

More than half 200 (52.1%), (95% CI 47.4–57.0%) of the 384 women had the intention to use LAPMs in health institutions of Aksum Town.

#### Factors associated with intention to use LAPMs

According to this study factors that were associated with intention to use LAPMs on bivariable analysis at the level of P-value 0.2 and less were fit into a multivariable logistic regression model. The multivariable analysis result of this study declared that knowledge on LAPMs, attitude on LAPMs, age, education, number of children desired, a decision on the number of children and the number of children alive had a statistically significant association with intention to use LAPMs (Table 3).

**Table 3:** Bivariable and multivariable analysis of associated factors of intention to use LAPMs of women in health institutions of Axum town, northern Ethiopia, March, 2016 (n = 384)

Variables	Intention to use LAPMs		OR	
	Yes	No	COR	AOR (95% CI)
	Number (%)	Number (%)		
Knowledge on LAPMs				
Low knowledge	65 (37.6%)	108 (62.4%)	1.00	1.00
Good knowledge	135 (64%)	76 (36%)	2.95***	2.15 (1.29, 3.56)**
Age of mothers				
18–24 years	59 (67.8%)	28 (32.2%)	2.37**	3.18 (1.30, 7.79)*
25–34 years	84 (47.7%)	92 (52.3%)	1.03	1.19 (0.60, 2.35)
≥ 35 years	57 (47.1%)	64 (52.9%)	1.00	1.00
Mothers’ education				
No formal education	27 (41.5%)	38 (58.5%)	0.57	0.70 (0.27, 1.80)
Primary school	31 (38.3%)	50 (61.7%)	0.49*	0.34 (0.14, 0.78)*
Secondary school	88 (62.4%)	53 (37.6%)	1.32	1.06 (0.50, 2.25)
Post secondary	54 (55.7%)	43 (44.3%)	1.00	1.00
Number of children desired				
0	32 (57.1%)	24 (42.9%)	1.28	10.21 (3.10, 33.58)***
1	45 (58.4%)	32 (41.6%)	1.35	4.70 (1.68, 13.13)**
2	65 (53.3%)	57 (46.7%)	1.10	1.95 (0.84, 4.52)
3	33 (41.2%)	47 (58.8%)	0.67	0.75 (0.32, 1.80)
4–6	25 (51%)	24 (49%)	1.00	1.00
Decision on the number of children				
Myself	23 (34.3%)	44 (65.7%)	1.00	1.00
Together	177 (55.8%)	140 (44.2%)	2.42**	2.05 (1.01, 4.18)*
Religion				
Orthodox	196 (53%)	174 (47.0%)	2.82	0.71 (0.18, 2.78)
Muslim	4 (28.6%)	10 (71.4%)	1.00	1.00
Attitude on LAPMs				
Negative attitude	40 (29.4%)	96 (70.6%)	1.00	1.00
Positive attitude	160 (64.5%)	88 (35.5%)	4.36***	3.41 (1.99, 5.85) ***
Heard myths and misconceptions				
Yes	131 (55.0%)	107 (45.0%)	1.37	1.06 (0.64, 1.74)
No	69 (47.3%)	77 (52.7%)	1.00	1.00
Household size				
1–4	141 (54.4%)	118 (45.6%)	1.34	0.22 (0.03, 1.70)
5–10	59 (47.2%)	66 (52.8%)	1.00	1.00
Number of children alive				
≤ 2	142 (54.6%)	118 (45.4%)	1.37	10.67 (1.29, 88.31)*
> 2	58 (46.8%)	66 (53.2%)	1.00	1.00
Mothers occupation				
House wife	100 (52.6%)	90 (47.4%)	0.74	0.46 (0.19, 1.07)
Private business	23 (41.1%)	33 (58.9%)	0.47	0.44 (0.16, 1.22)
Employed	50 (53.8%)	43 (46.2%)	0.78	0.53 (0.21, 1.35)
Unemployed	27 (60.0%)	18 (40.0%)	1.00	1.00

* Statistically significant at P < 0.05, ** statistically significant at P < 0.01, and *** statistically significant at P < 0.001; *OR* odds ratio, *AOR* Adjusted Odds Ratio, *COR* Crude Odds Ratio; no formal education includes illiterate and able to read and write

### Discussion

The finding of this study showed that 52.1% (95% CI 47.4–57) women reported that they had the intention to use LAPMs. This finding is in line with the study done in Adigrat town (48.4%) which is found in Northern Ethiopia [15].

It is a little higher in proportion to the study conducted in Debre Markos town, northwest Ethiopia (45.9%) [20]. This might be due to the study participants and design difference in both studies, in Debremarkos town only married women participated and the design was community-based whereas in this study the participants were women short-acting family planning users.

It is higher in proportion to the study conducted in Welita Zone, Southern Ethiopia (38%). This discrepancy could be explained by the difference in the study areas, and access to information and the services [16].

In this study, it was noted that knowledge of LAPMs was significantly associated with the intention to use LAPMs. Those women who had good knowledge of LAPMs were 2.5 times more likely (AOR 2.48; CI 1.55, 3.96) to have intention on LAPMs than women who had low knowledge of LAPMs. It was similar to the study conducted in Adigrat town [15]. This might be evident that knowledge is a prerequisite to intension.

Women aged 18–24 years were 5.3 times more likely (AOR 5.35; 95% CI 2.40, 11.91) to have the intention to use LAPMs compared to women whose age is ≥ 35 years. It was similar to the study conducted in Debre Markos town, northwest Ethiopia [20]. This could be because young women might get information on LAPMs better than the elder ones.

Women whose educational level post-secondary were about 2.4 times more likely (AOR 4.94; 95% CI 1.74, 14.01) to have intention than those women who didn’t attend formal education. It was in line with the study conducted in Nekemte town, Western Ethiopia [17], Wolaita Zone, Southern Ethiopia [16], Debre Markos town, northwest Ethiopia [20]. This implies that education has the pervasive impacts on women’s intention to use LAPMs since it empowers women with knowledge and positive attitude on contraceptive methods and hence, increases predisposition to their intention and use of LAPMs.

In this study, it was revealed that the number of children desired was significantly associated with the intention to use LAPMs (AOR 6.02; CI 2.10, 17.30). Women who had no desire for more children were six times more likely to have the intention to use LAPMs compared to those women who had the desire 4–6 more children. It was in line with the study conducted in Adigrat town, Northern Ethiopia [20]. This could be explained by the participants’ fear of fertility return after the use of long-acting methods. Not having a desire for more children was a significant predictor of intention to use contraceptives [18].

### Conclusion

In this study prevalence of intention to use LAPMs was low, 52.1%. The findings of this study showed that knowledge on LAPMs, attitude on LAPMs, age, education, number of children desired, decision on the number of children and numbers of children alive were significantly associated independent factors of the recent intention to use LAPMs among women who use short-acting contraceptive methods in health institutions of Aksum Town.

## Limitations

As the study design is cross-sectional, the identified factors for intention to use LAPMs might not precede the outcome intention to use.

## Supplementary information

**Additional file 1.** A questionnaire used to collect data on intention to utilize long acting contraception among women attending health institutions of Aksum town, Ethiopia.

## Data Availability

The data and materials used for analysis and conclusions are available in the manuscript as supplementary data.

## Abbreviations

AOR: Adjusted Odd Ratio
CI: confidence interval
FGAE: Family Guidance Association of Ethiopia
FP: family planning
LAPMs: long-acting and permanent contraceptive methods
SPSS: Statistical Package for Social Sciences
