# Supplementary material for: Intention to use long-acting and permanent contraceptive methods and associated factors in health institutions of Aksum Town, North Ethiopia
Source: BMC Res Notes. 2019 Nov 9;12:739. doi: 10.1186/s13104-019-4769-z (PMC6842538; doi:10.1186/s13104-019-4769-z)
Supplement: Supplementary file 1 — Additional file 1. A questionnaire used to collect data on intention to utilize long acting contraception among women attending health institutions of Aksum town, Ethiopia. [file 13104_2019_4769_MOESM1_ESM.docx]

# Annexes

## Annex I. Consent form in English language

I have been informed that the purpose of this study is to assess prevalence and associated factors of intention to use LAPMs. I have understood that participation in this study is entirely voluntarily. I have been told that my answers to the questions will not be given to anyone else and no reports of this study ever identify me in any way. I have also been informed that my participation or non-participation or my refusal to answer questions will have no effect on me. I understood that participation in this study does not involve risks.

Your signature below indicates that you have read or has been read to you the information provided above and have decided to participate in the study. If you later decide that you wish to withdraw from the study simply tell me, you may discontinue your participation at any time.

Signature of the participant: _____________ Date_______________

Time started: _______ Time finished: _________

If no, skip to the next participant

Interviewer Name_________________________Signature___________Date____________

Supervisor

Name_________________________Signature___________Date____________

#

## Annex II. English version Questionnaire

A questioner for intention to use long acting and permanent contraceptive methods among short acting contraceptive users in health institutions of Aksum town, northern Ethiopia

**Description of the questioner**

Questioner number: _______________________

Name of health institution: _____________________________________

**I. Socio-demographic characteristics**

| **No.** | **Question** | **Response** | **Skip** | **Code** |
| --- | --- | --- | --- | --- |
| 101 | Age | ____________ years |  |  |
| 102 | Ethnicity | Tigray ..............................1  Amhara ...........................2  Oromo .............................3  Other................................4 |  |  |
| 103 | Religion | Orthodox..........................1  Islam................................2  Protestant........................3  Catholic...........................4  Other...............................5 |  |  |
| 104 | Household size | _________ |  |  |
| 105 | Marital status | Married...........................1  Unmarried......................2  Divorced ........................3  Widowed .......................4  Separated .....................5 | If not married skip to  Q 107 |  |
| 106 | Husband's education | Illiterate...........................1  Read and write...............2  Primary...........................3  Secondary......................4  Post-secondary..............5 |  |  |
| 107 | Mother's education | Illiterate............................1  Read and write................2  Primary............................3  Secondary.......................4  Post-secondary...............5 |  |  |
| 108 | Mother's occupation | House wife......................1  Private business.............2  Private employ................3  Civil servant....................4  Unemployed....................5  Daily laborer....................6  Student............................7  Peasant...........................8  Other ...............................9 |  |  |

**II. Reproductive History**

| No. | Question | Response | Skip | Code |
| --- | --- | --- | --- | --- |
| 201 | History of abortion | Yes...................................1  No....................................2 | If no skip to Q203 |  |
| 202 | Number of abortions | _________ |  |  |
| 203 | Number of children alive | _________ |  |  |
| 204 | Desire for more child | Yes...................................1  No.....................................2 | If no skip to Q301 |  |
| 205 | Number of desired children | __________ |  |  |

**III. Knowledge and attitude on modern contraceptives**

| No. | Question | Response | Skip | Code |
| --- | --- | --- | --- | --- |
| 301 | Have you ever heard of LAPMs? | Yes........................................................................................1  No.........................................................................................2 | If no skip Q403 |  |
| 302 | Knowledge on LAPMs | Female sterilization .............................................................1  Male sterilization .................................................................2  Implants (Implanon/Jadelle/ Norplant)................................3  IUCD....................................................................................4  IUCD can prevent pregnancies for more than 10 years.....5  After female sterilization pregnancy is not possible...........6  The implant can prevent pregnancies for 3–5 years..........7  Vasectomy has no interference with sexual intercourse ...8  Implants require a minor surgical procedure during insertion and removal .......................................................................9  IUCD is not appropriate for female at high risk of getting STIs...................................................................................10 |  |  |
| 303 | Attitude on LAPMS | Using implants needs proper diet .........................................1  The insertion and removal of implants are highly painful.....2  Implant freely move in the body and cause severe pain.....3  Insertion of IUCD causes loss of privacy ............................4  Using IUCD restricts normal routine activities .....................5  Operation for female sterilization is dangerous...................6  For me, it is not good to use LAPMs....................................7  LAPMs not preventing one from having large family size...8  Discussing LAPMs with husband (friend) are not  Necessary............................................................................9  Husband decides if wife wants to use contraceptives.......10 |  |  |

**IV. Family planning information**

| No. | Question | Response | Skip | Code |
| --- | --- | --- | --- | --- |
| 401 | Intention to use LAPMs | Yes.........................................................1  No..........................................................2 |  |  |
| 402 | Method intended to use in the future | Implants.................................................1  IUCD......................................................2  Female sterilization...............................3 |  |  |
| 403 | What is the method that you are using currently? (short acting) | Injectable ...............................................1  Pills ........................................................2  Condom .................................................3  Others ....................................................4 |  |  |
| 404 | Decision on the number of children | Husband ...............................................1  Myself....................................................2  Together................................................3 |  |  |
| 405 | Heard myths and misconceptions | Yes........................................................1  No..........................................................2 | If no skip next? |  |
| 406 | What are the myths and misconceptions that you heard? | The implant would cause hypertension....1  Implant moves freely in the body and lost at the time of removal....................................2  Implant causes illness ..............................3  IUCD Causes illness.................................4  Implant causes anemia.............................5  IUCD causes anemia................................6  LAPMs cause infertility.............................7  Others .......................................................8 |  |  |

**Thank you for your time and valuable information!**
